# Fatal Attraction: The Case of Toxic Soluble Dimers of Truncated PQBP-1 Mutants in X-Linked Intellectual Disability

**DOI:** 10.3390/ijms22052240

**Published:** 2021-02-24

**Authors:** Yu Wai Chen, Shah Kamranur Rahman

**Affiliations:** 1 Department of Applied Biology and Chemical Technology, The Hong Kong Polytechnic University, Hunghom 999077, Hong Kong; 2 State Key Laboratory of Chemical Biology and Drug Discovery, The Hong Kong Polytechnic University, Hunghom 999077, Hong Kong; 3 Department of Infection Biology, London School of Hygiene & Tropical Medicine, London WC1E 7HT, UK; Shah.Rahman@lshtm.ac.uk

**Keywords:** Renpenning syndrome, intrinsically disordered protein, IDP, dimerisation, oligomerisation, misfolding, WW domain, aggregation, XLID

## Abstract

The frameshift mutants K192S^fs*7^ and R153S^fs*41^, of the polyglutamine tract-binding protein 1 (PQBP-1), are stable intrinsically disordered proteins (IDPs). They are each associated with the severe cognitive disorder known as the Renpenning syndrome, a form of X-linked intellectual disability (XLID). Relative to the monomeric wild-type protein, these mutants are dimeric, contain more folded contents, and have higher thermal stabilities. Comparisons can be drawn to the toxic oligomerisation in the “conformational diseases”, which collectively describe medical conditions involving a substantial protein structural transition in the pathogenic mechanism. At the molecular level, the end state of these diseases is often cytotoxic protein aggregation. The conformational disease proteins contain varying extents of intrinsic disorder, and the consensus pathogenesis includes an early oligomer formation. We reviewed the experimental characterisation of the toxic oligomers in representative cases. PQBP-1 mutant dimerisation was then compared to the oligomerisation of the conformational disease proteins. The PQBP-1 mutants are unique in behaving as stable soluble dimers, which do not further develop into higher oligomers or aggregates. The toxicity of the PQBP-1 mutant dimers lies in the native functions (in transcription regulation and possibly, RNA splicing) being compromised, rather than proceeding to aggregation. Other examples of stable IDP dimers were discussed and we speculated on the roles of IDP dimerisation in protein evolution.

## 1. Introduction

Structural biologists have long recognised that proteins often contain disordered regions. Around 1990, there was enough experimental evidence of these unstructured regions, which challenged the conventional protein structure-function paradigm [1]. As more examples of these emerged, it was soon acknowledged that substantial regions, domains or even whole proteins can be without a folded structure in physiological conditions. These unstructured regions are now referred to as “intrinsically disordered” regions or proteins (IDRs or IDPs). Unlike those of a folded protein, the peptide backbone torsion angles of IDRs undergo segmental dynamic switching which results in the fast exchange of many conformation states [2]. IDRs and IDPs are common in eukaryotic proteomes [3]. In mammals, half of their total proteins are predicted to contain IDRs of >30 residues, and approximately a quarter of their proteins are predicted to be fully disordered [4]. For a recent comprehensive review, see [5]. The reader is also referred to this review for an attempt on the classification of IDPs [6].

From a functional perspective, IDPs can bind promiscuously to a wide range of targets. They usually play the important role of hubs in a complex interaction network. Comparing with folded proteins, the binding of IDPs towards their partners is of low affinity but specific [7], thus they are well suited for regulatory reversible interactions.

IDPs are implicated in many human diseases when the delicate balance of order and disorder was disrupted due to environmental and concentration changes [3], dysregulation of protein modification [8] or mutations [9]. The IDR part of the proteins or an IDP may become misfolded and lead to unfavourable structural consequences, which are frequently manifested as aggregation. Researchers refer to these diseases collectively as “conformational diseases” since the pathogenesis involves a substantial change in protein structure. Many of these disorders affect the nervous system, including the prion diseases, Alzheimer’s disease, Parkinson’s disease, and polyglutamine (polyQ) expansion diseases (e.g., Huntington’s disease). The genes and proteins implicated in all these conformational diseases are unrelated, yet extensive experimental works converge to a general pathogenesis, with some variations [10]. It was revealed that at the early stages of these diseases, monomeric proteins with IDR or IDPs form soluble oligomers which seed further protein-protein interactions on a massive scale to form aggregates [11,12,13,14,15,16,17,18]. The oligomers are thus considered to be the “toxic” intermediates paving the irreversible way to form insoluble polymers.

In this review, we focused on the oligomerisation events of IDRs or IDPs, and their importance in human neuronal diseases. In this context, we briefly discussed representative works on the detection and characterisation of the toxic oligomers in the conformational diseases. The subsequent aggregation process was outside the scope of this review. Due to the difficulties in sample collection and preparation, and different experimental requirements (e.g., time scale of protein aggregation), one would often find inconsistent or even contradicting reports. Next, we switched to some earlier structural work on the polyglutamine tract-binding protein 1 (PQBP-1) mutants of our laboratory and discussed the implications of their dimerisation in X-linked intellectual disability (XLID). We compared the cases of the PQBP-1 mutants with the toxic oligomerisations in the conformational diseases and highlighted the uniqueness of the former. Finally, we introduced several other examples of physiological IDP dimerisation and also speculated on a possible role of IDP dimerisation in protein evolution.

## 2. Folding and Misfolding Energy Landscape

**Figure 1:**
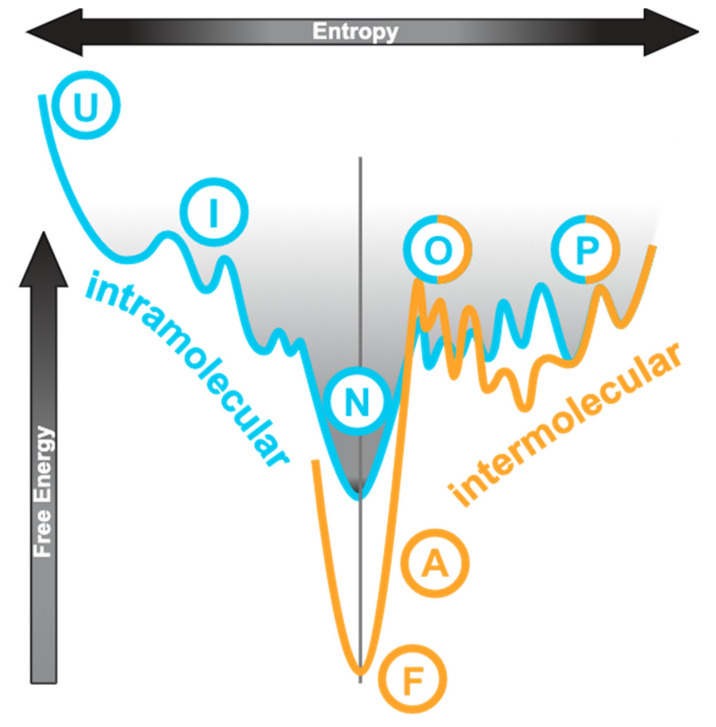
Conceptual relationship of conformational states involved in diseases. This shows a cross-section through an energy funnel with entropy as the horizontal axis and free energy as the vertical axis. The left half of the cyan curve represents a generic folding via intermediate path from unfolded (U), (I), to native (N) states. For intrinsically disordered proteins (IDPs), the native state is partially folded (P) or can easily become so since the free energy difference is small and it is characterised by multiple states including the oligomers, as illustrated by the right half of the cyan curve. The orange curve to the right is the misfolding funnel, also with the partially folded (P) and oligomeric (O) states. In diseases, a structural transition occurs in either the partially folded state or the oligomeric state, leading to stable amorphous aggregates (A) or fibrils (F). The normal folding pathway is dominated by intramolecular contacts whereas the misfolding pathway by intermolecular interactions. This figure is adapted and modified from this source [22]. Moreover, see this for a discussion of the IDP energy landscape [16].

The conventional protein folding pathway can be represented by an energy profile as shown in Figure 1, on the left-hand side of the vertical axis. The unfolded state (“U”) is high in both entropy and free energy. Folding is a process by which the molecular species achieves the lowest free energy state (native or folded, “N”) and has its overall entropy reduced, which usually progresses via an intermediate state (“I”). The process is generally driven by intramolecular interactions including hydrophobic contacts, hydrogen bonding, and electrostatic interactions. An IDP has a very different energy landscape?it has multiple unfolded or partially folded states (“P” in Figure 1) that do not differ much in free energy and thus transitions between these states are transient and reversible [16,17]. This is illustrated by the cyan curve to the right of the axis in Figure 1. Within these disordered states, the extent of compactness and residual structures may vary leading to descriptions such as “molten globule” and “pre-molten globule” being used [6]. IDPs do not have a distinguishable lowest free-energy native state.

Some molecular species among the heterogeneous IDP population can occasionally be promoted into the oligomeric states which differ little in free energy (Figure 1, cyan curve to the right of axis, “O”). The oligomeric states can then progress to the formation of stable amorphous aggregates or fibrils (Figure 1, orange curve, “A” and “F”, respectively) which are low in free energy. The process is irreversible and is known as misfolding. The partially folded monomer (“P”) and the oligomer (“O”) states are shared by both the IDP energy landscape and the misfolding landscape. From oligomerisation onwards, energy consideration is dominated by intermolecular interactions. When an IDP binds to a target, its interaction interface may remain disordered (“fuzzy binding” [6,19,20,21]) or become ordered (“induced fit”) [6,7,16]. “Induced fit” refers to the IDP binding to its target as a fully unfolded species followed by folding in situ to form secondary structures. Here, we do not differentiate “conformational selection”, which describes the IDP as an ensemble of extended conformations with residual structures from which the best one is favoured by the binding target, from “induced fit”.

## 3. Oligomers in the Conformational Diseases

Extensive works on the prion diseases have laid the foundation to develop an “infection-replication” model which serves as a prototypical mechanism to interpret the findings on the other conformational diseases [23]. In this section, the works on the early oligomerisation events and on characterising the toxic oligomers of these disease proteins will be described. It must be stressed that the detailed schemes are far from clear or complete. Different experimental executions often dictated the findings. Despite the fact that there are controversies, however, the general pathogenesis that is common to all conformational diseases has been emerging.

The cellular prion protein (PrP^C^) is soluble and partially unfolded, with a globular C-terminal domain that consists of three α-helices and one short sheet. PrP^C^ undergoes a very fast (sub-millisecond) structural transition, which eventually leads to the formation of insoluble amyloid fibrils of PrP^Sc^ (the “scrapie” pathogenic isoform, a reference to the mad cow disease) that are predominantly β-structures [23]. However, it was demonstrated that prion propagation is biphasic and that yet another isoform, PrP^L^ (for lethal) is the neurotoxic (uncoupled from infective) species [24,25,26]. Structural studies on the misfolded oligomer recorded several helix-to-strand conversions in the C-terminal folded domain, rather than in the N-terminal IDR [27]. With hydrogen-deuterium exchange mass spectrometry (HDX/MS), some insight was gained about the early partially folded monomeric intermediate [23]. The critical step of pathogenesis involves a structural transition step to form the soluble misfolded oligomers, and it was found that oligomers of 14~28 units were the most infectious [28]. The study of disease mutants G113V and A116V, both in the IDR, showed that dimerisation is the rate-limiting step of oligomerisation [29].

In Alzheimer’s disease, the emergence of neurofibrillary tangles from aggregated tau proteins is a pathological hallmark [8,13,30,31,32,33]. The accumulation of tau results in insoluble depositions in neurons and a broad range of neurodegenerative conditions with different extents of clinical symptoms. While tau exists in many different forms from monomer to dimer/trimer, soluble oligomers, granular higher oligomers (~40 monomers), tangles, filaments and amorphous aggregates, it is most likely that the small oligomers are the toxic species [15]. However, it is known that a compact monomeric form with intramolecular disulphide bonds is non-toxic [15]. When the concentration of the protein increases, the disordered tau monomer can readily adopt the partially folded states which are detectable by virtue of their β-contents [15]. Helped by disulphide bond formation, the β-rich partially folded monomer dimerises easily [15]. The development of antibodies which are specific for the tau oligomers (dimers or trimers) made it possible to monitor the early events [34,35,36]. In one study, researchers used single-molecule fluorescence methods to monitor the process of monomers turning into small oligomers which then undergo a slow structural conversion before fibrillation [37]. Hyper-phosphorylation and many environmental factors were found to affect the rate of tau aggregation, but the initiation step remains elusive [15]. Analogous to the case of PQBP-1 (see later), the removal of its C-terminal domain also accelerates tau polymerisation [38,39].

Amyloid-β proteins (Aβ_1–40_ and Aβ_1–42_) are monomeric IDPs also implicated in Alzheimer’s disease. The unstructured monomers assemble into fibrils that are rich in β-contents. The fibrillation process was followed with circular dichroism (CD) spectroscopy and showed a bell-shaped distribution of helical contents over time before the sample became predominantly β-structures [40]. The researchers suggested that a structural transition occurred when monomers formed the oligomeric species and estimated that the number of monomers in the helix-rich oligomer was ~23 [40]. A similar study, however, reported that the oligomer was a predominantly hexamer with the unusual “α-sheet” secondary structure which has no CD signature [41]. It was a breakthrough when two structures of Aβ_1–42_ oligomers were solved by NMR spectroscopy: One was a tetramer and the other an octamer [42]. The tetramer showed a six-stranded β-sheet arrangement, whereas the octamer was a β-sandwich structure with two stacking tetrameric-sheets. Molecular dynamics simulations demonstrated that both oligomers may form channels crossing a lipid bilayer, offering one possible molecular mechanism of membrane damages in neurons [42].

α-Synuclein is a monomeric IDP which turns into aggregates in Parkinson’s disease [16,43,44,45]. However, there were other reports claiming that the samples existed as a soluble folded tetramer with helical content [46,47], with thermal denaturation behaviour typical of a folded protein [47]. The Parkinson’s disease mutants A30P, A53T, and E46K of α-synuclein showed spectra typical of disordered proteins [47]. At lower concentrations (<0.5 mg mL^−1^), α-synuclein was disordered [47]. The behaviour of the protein is best described by a concentration-dependent equilibrium between the disordered monomer and the folded tetramer [47,48]. In this case, the soluble tetramer was stable and resisted aggregation, therefore it was not the toxic oligomer which primed fibril formation (protofibril) [48,49]. In other reports, many species of oligomers have been identified, of varying sizes up to ~30 units, with predominantly β-structures [49]. Recent research suggested that an interaction with lipids may trigger the formation of the toxic oligomer [50]. α-Synuclein cellular pathology can propagate among neurons with a prion-like seeding mechanism and it was speculated that the toxic oligomers are the “infectious” agent that mediates the transmission [48].

PolyQ expansion diseases consist of nine inherited neurodegenerative disorders (for a recent review, see [51]). In each case, the protein harbours a glutamine repeat tract the size of which is enlarged in disease sufferers due to CAG triplet repeat expansion in the respective gene. Proteins with an expanded polyQ tract deposit as insoluble fibrils inside neurons. Using fluorescence correlation spectroscopy in cells, a soluble β-rich monomer of expanded-sized (pathogenic) polyQ peptide (without a protein context) was detected before amyloid formation [52], and the soluble monomer formed massive aggregates via oligomeric (dimers or trimers) intermediates [53]. The soluble β-rich toxic oligomers were detected before the formation of insoluble fibrils [53,54]. Full-length huntingtin protein (implicated in the Huntington’s disease) with a pathogenic-sized glutamine repeat tract was produced from the cell culture successfully. It consisted of mainly monomeric (36%) but also stable dimeric (19%) and trimeric proteins (17%) [55]. However, the purified mutant huntingtin has no noticeable difference in the overall secondary structure contents relative to a sample having a non-pathogenic-sized (wild-type) tract [55]. In the cell culture, the mutant huntingtin exon 1 transcript (a proteolytic product of huntingtin N-terminal domain which contains a pathogenic polyQ tract) monomer was observed to accumulate to form small oligomers (5~15-mers), followed by nucleation and formation of aggregates [56]. Another study reported a similar result, only that the monomer was absent, and the smallest species observed was tetrameric [57].

To sum up, in each of the above diseases, researchers identified toxic oligomeric species: Some are stable, others are transient. The number of monomers in these oligomeric forms can vary from two up to low tens. They are toxic in the sense that they become seeds of aggregation, i.e., they are the agents that propagate the disease condition. In some cases, the structural transition was detected before oligomerisation, but this was not consistently observed.

## 4. Polyglutamine Tract-Binding Protein 1 (PQBP-1)

While looking for proteins which interact specifically with the polyQ tract, Okazawa et al. isolated a protein and named it the polyglutamine tract-binding protein 1 (PQBP-1) [58]. The expression pattern of PQBP-1 suggested its importance in early brain development. The mutations in PQBP-1, mostly frameshifts, have deleterious effects that lead to severe cognitive impairment and results in the Renpenning syndrome, a type of X-linked intellectual disability (XLID) [59]. Its Y65C mutation leads to the Golabi-Ito-Hall syndrome which is another XLID disorder [60].

**Figure 2:**
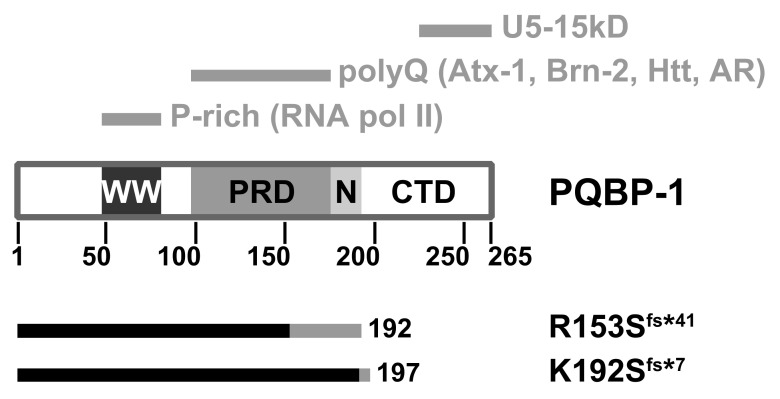
Domain architecture of the polyglutamine tract-binding protein (PQBP-1). WW: WW domain; PRD: Polar amino-acid-rich domain; N: Nuclear localisation signal; CTD: C-terminal domain. The top grey bars indicate the respective regions which mediate molecular interactions. The bottom bars indicate the respective truncation mutants: The native sequence is in black, whereas the non-native (due to frameshift) is in grey. Atx-1: Ataxin-1; Htt: Huntingtin; AR: Androgen receptor.

Analysis of the amino acid sequence of PQBP-1 shows it contains three domains (Figure 2). The WW domain (residues 48-81) is a small three-stranded antiparallel β-sheet structural motif commonly found in signalling and transcription regulation components [61,62]. This is the only folded part which mediates its interaction with RNA polymerase II [58,63] and also binds the splicing factor WBP11 [64]. The polar aimno-acid-rich domain, PRD (residues 104-163) is a highly charged region which binds to glutamine repeat tracts [65]. The C-terminal domain (CTD) contains a conserved YxxPxxVL motif within residues 223–265 that interacts with the spliceosomal protein U5-15kD [65,66,67] and the splicing factor TXNL4A [68].

### Structural Changes of Mutants

We studied the mutant proteins implicated in two particularly severe clinical manifestations [69]: A four-basepair deletion (c.459_462delAGAG) which produces a 192-residue mutant protein, p.Arg153Serfs*41 (denoted R153S^fs*41^ here); and a two-basepair deletion (c.575_576delAG) that produces a 197-residue mutant protein, p.Lys192Serfs*7 (denoted K192S^fs*7^) [59,70,71]. Both frameshift mutants are shortened by approximately 25% compared to the wild type (Figure 2).

**Figure 3:**
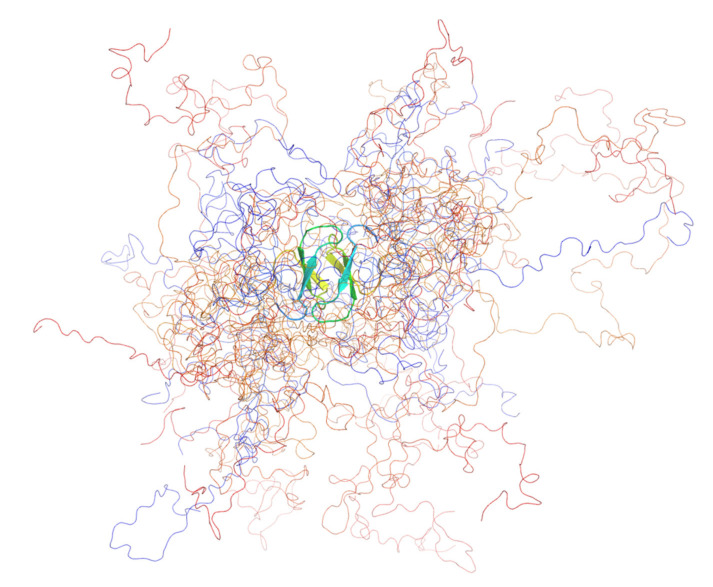
The ensemble model of PQBP-1 K192S^fs*7^ containing 12 structures. The ensemble was optimised by fitting to the solution scattering data. The protein was coloured in a rainbow spectrum, from blue (N-terminus) to red (C-terminus). All the models shared a common dimeric WW domain, which was shown in the cartoon representation, whereas the rest of the disordered protein was shown as coils. This figure was produced with PyMOL.

We were surprised to find that, compared with the monomeric wild type [72], both mutants were dimeric, as demonstrated conclusively by sedimentation analytical ultracentrifugation (AUC) and small-angle X-ray scattering (SAXS). From the SAXS analysis (Kratky plots), we observed that the two mutants were substantially more compact and more folded than the wild-type [69], granted, that PQBP-1 is natively unfolded [72,73]. Both mutants were considerably more stable than the wild-type protein with the dimer melting to the unfolded state with an intermediate (in the case of R153S^fs*41^) or without one (K192S^fs*7^). Wild-type PQBP-1 has a free energy of folding, *ΔG* of −2 kJ mol^−1^, which is relatively small and is typical for IDPs [69]. The *ΔG* of K192S^fs*7^ was measured to be −32 kJ mol^−1^; and the overall *ΔG* of R153S^fs*41^ was measured to be −59 kJ mol^−1^ (two steps: *ΔG*_1_ = −25 kJ mol^−1^; *ΔG*_2_ = −34 kJ mol^−1^) [69]. Backbone structural models of the two mutants were produced using an ensemble optimisation method (EOM) (Figure 3) [69].

From the solution scattering data, we observed some signs of aggregation when the sample concentrations reached 7 or 9 mg mL^−1^. The extent of aggregation was not severe as the Guinier analyses could be completed by omitting the few lowest-angle data (unpublished). With sedimentation AUC experiments, K192S^fs*7^ at 1.4 mg mL^−1^ showed some higher oligomer (4–6 monomers) formation. No high-molecular-weight aggregates were detected for either mutant [69].

## 5. Toxic Dimerisation

### 5.1. The Uniqueness of the PQBP-1 Toxic Dimer

As discussed in Section 3, the IDPs form transient oligomers, hardly detectable in the extreme cases, which then seed irreversible aggregation or dissociate back into the IDP monomers [10]. A structural transition sometimes accompanies the oligomer formation. This general scheme is represented by:

*n* U^1^ ⇌ (F^1^)*_n_* → (F^1^)_∞_ (1)

where U^1^ stands for the unfolded species (IDP) 1 and F^1^ stands for the (partially)-folded conformation of species 1.; *n* is a small integer and ∞ represents a large number.

In the case of PQBP-1, the wild-type protein is a monomeric IDP. Its truncated disease mutants form stable (partially)-folded *homodimers* that are non-functional, as represented by:

2 U^1^ → (F^1^)_2_ (2)

It has been established that some IDPs show induced folding on binding their folded targets—but these complexes are heterodimers:

U^1^ + F^2^ ⇌ F^1^:F^2^ (3)

The general misfolding disease scheme with the description of a toxic oligomeric intermediate was reviewed comprehensively (Figure 1) [16,17,21]. In the two PQBP-1 XLID disorders, analogous toxic dimers are detected, yet the dimers do not progress to aggregation. The increase of structural contents, relative to the natively disordered state, led to this phenomenon being coined the term, “mis-ordering” [17].

### 5.2. Dimerisation as a Means of Regulation

Why does the truncation of the C-terminal region in the PQBP-1 mutants lead to dimerisation? Since the N-segment (residues 1–219) of PQBP-1 interacts with the C-segment (residues 220–265) [74], it seems plausible that the CTD has an auto-inhibitory function against homo-dimerisation [69]. Further, it is likely that the intermolecular interactions mediated by the CTD [66] also disfavour homo-dimerisation. We proposed that the WW domain is involved in forming the dimeric interface [69]. In the other PQBP-1 disease (Golabi-Ito-Hall syndrome), dimerisation of PQBP-1 was speculated to be promoted by the missense mutation (Y65C) in the WW domain [75,76,77]. Folding-wise, PQBP-1 was best described by the molten globule state, consisting of near-native secondary structures with a loosely packed hydrophobic core [72,78]. The shallow melting curves of PQBP-1 and the two mutants indicated that their “folding” represented an increase of compactness of the molten globular states [69] and the interface was best described as a fuzzy interaction. Two effects might account for the observed increase in the structural content of both PQBP-1 mutants. First, it was a result of the deletion of the largely disordered C-terminal region [73,74]. Second, it was conceivable that two intrinsically disordered molecules mutually induce some residual secondary structures when they were in close proximity. With this, we speculate that the equilibrium between the active monomer and the inactive dimer may even be a negative feedback mechanism of regulation of normal PQBP-1 functions. This is the opposite way of regulation by an equilibrium between the inactive monomer and the active dimer observed in the epidermal growth factor receptor (EGFR) signalling pathway [79].

### 5.3. Loss of Function

To study the role of PQBP-1 in transcription, we investigated its binding to a representative phosphorylated heptapeptide (YpSPTpSPS) representing the C-terminal domain of RNA polymerase II, which consists of 52 repeats of these [80]. Wild-type PQBP-1 bound the peptide with a dissociation constant, *K*_d_ of 154 μM. The mutants K192S^fs*7^ and R153S^fs*41^ bound substantially weaker with *K*_d_ values of 456 and 338 μM, respectively [69].

The K192S^fs*7^ mutant is in effect likened to PQBP-1 lacking its C-terminal region, whereas R153S^fs*41^ has a disrupted PRD followed by a long non-native tail. The loss of the CTD in most of the PQBP-1 frameshift mutants will abolish its interactions to the spliceosomal protein U5-15kD and factor TXNL4A [66,68]. Non-native associations with other cellular molecules may also be present due to the higher stabilities of the mutants. For R153S^fs*41^, it may also interact non-natively with cytoplasmic molecules [69] since its nuclear localisation is compromised [70].

The term “toxic oligomers” was conventionally used to reflect the infectious nature of the soluble species which promote aggregation or fibril formation [43]. In the case of PQBP-1 mutants, the dimers are toxic in the true sense that they lose or have reduced native functions.

## 6. Implications

### 6.1. Other Stable Non-Toxic Dimers

The transcription factor Max is a famous early example described by scheme (2), Section 5.1. Max is in equilibrium between a monomeric unfolded state and a homo-dimeric leucine-zipper folded state [81,82]. Sigalov et al. have reported the formation of specific IDP dimers in the immune receptors [83]. The main difference between the PQBP-1 mutant dimers and the immune receptor dimers was the lack of increased structure in the latter. The monomeric and dimeric IDP components had identical structures in the immune receptors [83].

The ribonucleotide reductase inhibitor Sml1 is a yeast IDP which exists in a dynamic equilibrium between the monomeric and dimeric states. No induced structure was observed on dimerisation [84]. Similarly, reduced granulin-B is also maintained in a monomer-dimer dynamic equilibrium, with no gain in structure on dimerisation [85]. Further, it was found that the protein was active only at low concentrations (<1 μM)—very similar to the monomer-dimer control we speculated in PQBP-1 (Section 5.2).

The mammalian HMGA2 is another example of an IDP which forms stable homo-dimers [86]. In this case, the interface was mediated mainly by electrostatic interactions bridging the highly charged “AT-hooks” and the C-terminal regions.

The above examples illustrated that IDP dimerisation is not uncommon, and usually, the interface remains fuzzy.

### 6.2. On Protein Evolution

The conventional wisdom is that the structure confers a negative impact on molecular evolution [7,87,88,89,90]. Another way to look at it is that IDRs are more generous in accommodating mutational side effects. A recent study examining the multimerisation of proteins made an insightful discovery, that protein oligomerisation does not necessarily confer immediate functional advantage, instead, the oligomerisation event creates opportunities for a subsequent acquisition of new or modified functions that are otherwise inaccessible to the monomeric state [91]. This was exemplified by the enrichment of hydrophobicity at the buried dimer interface. The implication is that oligomerisation may expand the evolutionary space of monomeric proteins. The cases of PQBP-1 mutations are detrimental to the host so they do not qualify as useful intermediates along the path of evolution. However, there is a 224-residue natural splicing variant named PQBP-1b/c, the sequence of which is very similar to the K192S^fs*7^ mutant [69,92]. There is no biophysical information on this protein, but we speculate, based on our work, that PQBP-1b/c is likely to be a dimer since it has the CTD replaced by a short tail. If this is true, then the (PQBP-1b/c)_2_ dimer with induced folding at the dimeric interface can be a potential framework on which new functions can be crafted.

## 7. Conclusions

We described the biophysical studies of two PQBP-1 XLID mutant proteins and contrasted their pathogenic mechanisms with those of the misfolding diseases. The PQBP-1 diseases are unique in the sense that the soluble dimeric species are the stable end states, which are toxic in nature with loss of functions and possibly toxic gain of functions. For the conformational diseases, it was speculated that the soluble oligomer may be a target for pharmaceutical development. If an inhibitor can be developed against oligomerisation, or preferentially stabilise the native non-toxic oligomeric form (in α-synuclein) of the protein target, the irreversible misfolding can be halted [48]. A similar approach was used in screening small molecules (curcumin derivatives) against tau oligomerisation [93]. In the cases of the PQBP-1 mutants, it is conceivable that such an approach may be used to identify agents which can rescue the functions that are lost or reduced, using a transcription activity assay. However, the functions lost by the deletion of the CTD may not be easily compensated.

## Abbreviations

PQBP-1	polyglutamine tract-binding protein 1
XLID	X-linked intellectual disability
IDR/P	intrinsically disordered region/protein
polyQ	polyglutamine
PrP	prion protein
Aβ	amyloid-β protein
PRD	polar amino-acid-rich domain
CTD	C-terminal domain
HDX/MS	hydrogen-deuterium exchange mass spectrometry
CD	circular dichroism
NMR	nuclear magnetic resonance
SAXS	small-angle X-ray scattering
AUC	analytical ultracentrifugation
EOM	ensemble optimisation method
